# Paternity Analysis of the Olive Variety “Istrska Belica” and Identification of Pollen Donors by Microsatellite Markers

**DOI:** 10.1155/2014/208590

**Published:** 2014-07-06

**Authors:** Alenka Baruca Arbeiter, Jernej Jakše, Dunja Bandelj

**Affiliations:** ^1^Science and Research Centre, University of Primorska, Garibaldijeva 1, 6000 Koper, Slovenia; ^2^Agronomy Department, Biotechnical Faculty, University of Ljubljana, Jamnikarjeva 101, 1000 Ljubljana, Slovenia; ^3^Faculty of Mathematics, Faculty of Mathematics, Natural Sciences and Information Technologies, University of Primorska, Glagoljaška 8, 6000 Koper, Slovenia

## Abstract

The leading olive variety in Slovenia is “Istrska belica” (*Olea europaea* L.), which currently represents 70% of all olive trees in productive orchards. Paternity analysis based on microsatellite markers was used for genotyping and identification of the potential pollen donors of “Istrska belica” and for assessing the proportion of self-fertilization in monovarietal olive orchards in the Slovene Istria. Seven microsatellite loci were used for genotyping thirty-one olive embryos from “Istrska belica” trees and for all potential pollen donor varieties, which are grown in the region and could participate as pollinators. Genotyping results and allele identification were performed using the FaMoz software. The most probable pollen donor was assigned to 39% of all analyzed embryos. Among all analyzed embryos no single case of self-fertilization was confirmed. According to the present results, the variety “Istrska belica” was in all cases fertilized by foreign pollen. The results will contribute to defining the new guidelines for farmers regarding the proper management and growing practice in monovarietal olive groves.

## 1. Introduction

The olive (*Olea europaea* L.) is a long-living evergreen oil fruit tree. Its cultivation is widely spread in the Mediterranean Basin, which provides about 90% of the world olive production [1]. Favorable nutritional properties of olive oil contributed to the increasing consumption worldwide, and thus the olive production has recently started in countries with similar Mediterranean climate conditions such as Argentina, Chile, Mexico, the US, New Zealand, Australia, and South Africa [2].

The extraordinary regenerative capacity of olive trees, compatibility and crossing between wild, feral, and cultivated forms, and fixation of desirable olive genotypes through vegetative propagation [3] have contributed to the diversity of olive varieties. There are more than 1,000 olive varieties under cultivation, which have originated from selections made by growers over many centuries [4]. The choice of varietal structure for the growing region significantly contributes to the quality of olive oil, and compatibility of varieties is one of the most important factors affecting the amount and durability of the yield that should be considered for the selection of varieties in olive orchard.

Although the olive flowers are hermaphroditic and wind pollinated, most varieties are self-incompatible which means that the flowers cannot be fertilized by pollen from the same variety and need other varieties as pollen donors. The choice of varieties and their pollinators thus determines the fertility, fruit set, and consequently the productivity of olive orchards, which are all important factors for successful and economic production.

Traditional approaches for studying compatibility of olive varieties include artificial pollination and crossing [5], isolation of flowers using pollination bags [6], pollen tube growth observation [7], and measurement of the fruit set [8]. The successes of the mentioned experiments are strongly influenced by environmental, weather, and seasonal conditions and have often led to the conflicting results [9–11].

With development of molecular markers, a quick and reliable method for pollen donor identification became available. Microsatellites are probably the most efficient markers for paternity testing, due to their Mendelian segregation, codominant nature, and hypervariability. The usefulness of microsatellites in paternity tests for assessment the self-compatibility and identification of compatible pollinizers of the olive variety “Kalamata” has already been demonstrated [12]. The pollination patterns in commercial olive groves with use of microsatellites have been also investigated in Australia [13, 14]. In Spain the paternity of olive seeds from defined crosses in breeding programs has been performed in order to assess the degree of pollen contamination [15].

In Slovenia the potential for olive cultivation is limited to the areas near the Adriatic coast. The varietal structure of olives has shaped over the centuries and is a result of different attempts to revitalize olive growing in the region affected by severe frosts. In the last thirty years, there has been a general trend of increased olive planting in Slovenia, and, at present, approximately 700 tons of olive oil is produced annually on some 1,900 hectares of land. The leading variety in Slovenian olive orchards is “Istrska belica” which represents 70% of olive trees in the region. This variety was intensively propagated in the 20th century in the Slovene Istria region [16] and Friuli Venezia Giulia region (Italy) [17], due to its adaptability to low temperatures, very good and regular yield, and high biophenols and oil content. “Istrska belica” usually gives ample and regular crops in well-maintained plantations. Nevertheless, some growers have reported problems with fertility and fruit set. Contrasting information about the compatibility of this variety is published in the literature. According to some authors [18, 19] the variety is self-compatible, while in the World Catalogue of Olive Varieties, IOOC [20] is determined as partly self-compatible. The aim of the present study was to identify pollen donors for the olive variety “Istrska belica” by paternity analysis using microsatellite markers and to assess the proportion of self-fertilization in monovarietal olive orchard.

## 2. Materials and Methods

### 2.1. Plant Material and DNA Extraction

Sampling of olive fruits was performed in monovarietal olive orchard in Osp, Slovene Istria, at full maturity in November 2011. For paternity test, ten fruits from four olive trees of the variety “Istrska belica” were collected. Fruits were harvested from four canopy segments (north, south, east, and west). Altogether 40 olive fruits were included in analysis.

DNA was extracted from each olive embryo, the mesocarp was removed from the olive fruit, and the endocarp was cracked with a vice. The diploid embryo was separated from the endosperm using a pair of forceps and DNA was extracted by modified method developed by Guerin and Sedgley [14]. The individual embryo was immersed in 500 *μ*L of grinding buffer (100 mM TRIS-HCl, pH 8.0, 20 mM EDTA, and pH 8.0, with 4 mg/mL diethyl dithiocarbamic acid sodium salt added just before use) in a 2 mL microcentrifuge tube. The embryo was ground with the buffer and kept on ice until all the samples were ready. Then the samples were incubated for 10 min at 65°C followed by the addition of 500 *μ*L of lysis buffer (100 mM TRIS-HCl, pH 8.0, 20 mM EDTA, pH 8.0, with 1 M NaCl, 2% SDS, and 1% sodium metabisulphite added just before use) and incubated for another 30 min at 65°C. The tubes were inverted every 10 min during this step. Afterwards, the tubes were cooled on ice and 1 mL of phenol : chloroform : isoamyl alcohol (25 : 24 : 1) was added. The tubes were mixed and centrifuged for 20 min at 14.000 rpm and the supernatant was removed to a fresh 1.5 mL tube. 500 *μ*L of ice-cold isopropanol was added to each tube and kept on ice for 1.5 hour. The tubes were centrifuged for 15 min at 14.000 rpm and the supernatant was removed. The pellets were washed with 1 mL of 76% ethanol. The supernatant was then decanted and the DNA pellets were dried at room temperature. Pellets were suspended in 50 *μ*L of TE buffer (10 mM TRIS-HCl, pH 8.0, 1 mM EDTA, and pH 8.0). DNA concentrations were measured by a Qubit fluorometer (Invitrogen).

For the identification of potential pollen donors of variety “Istrska belica”, twenty-six microsatellite profiles of reference olive varieties (“Arbequina,” “Ascolana tenera 1,” “Ascolana tenera 2,” “Athena,” “Belica Pucer,” “Buga,” “Cipressino,” “Coratina,” “Črnica,” “Frantoio,” “Grignan,” “Itrana,” “Leccino,” “Leccio del corno,” “Leccione,” “Maurino,” “Moraiolo,” “Nocellara del Belice,” “Pendolino,” “Picholine,” “Samo,” “Samo Nova vas,” “Santa Catarina 1,” “Santa Caterina 2,” “Štorta,” and “Zelenjak viseča”) from a national genotyping database were included in the analysis.

### 2.2. Genotyping Procedure

Seven microsatellite loci were included into the paternity test: ssrOeUA-DCA3, ssrOeUA-DCA9, ssrOeUA-DCA11, ssrOeUA-DCA14, ssrOeUA-DCA16 [21], UDO99-019 [22], and EMO-3 [23]. Amplification of microsatellites was carried out in PCR reactions in a total volume of 15 *μ*L, containing 1x supplied PCR buffer (Promega, Manheim, Germany), 2 mM MgCl_2_, 0.2 mM of each dNTP (Sigma-ALDRICH, St. Louis, USA), 0.2 *μ*M of each locus specific primer with one of the primers in pair elongated for M13(−21) universal sequence [24], 0.25 *μ*M of M13(−21) universal primer labelled with FAM, VIC, PET, or NED (Applied Biosystems), 0.375 unit of Taq DNA polymerase (Promega, Manheim, Germany), and 20 ng of olive DNA. The amplification was performed on a Thermal Cycler 2720 (Applied Biosystems), and the conditions of the two-step PCR were as follows: 94°C (5 min), then 5 cycles for 45 s at 94°C, 30 s at the initial annealing temperature of 57°C, which was lowered by 1°C in each cycle, and the extension at 72°C for 1 min 30 s. The second step of amplification passed through 25 cycles of 30 s at 94°C, 30 s at the initial annealing temperature of 52°C, and 1 min 30 s elongation at 72°C, followed by a final extension step of 8 min at 72°C. Separation of amplified microsatellites was performed on automatic ABI 3130 (Applied Biosystems) sequencer and data were analyzed using Gene Mapper 4.1 (Applied Biosystems) software.

### 2.3. Data Analysis

Based on genotyping data with microsatellites, the following parameters of genetic variability and information content were calculated for the offspring population (embryos) and for the parental population (26 reference olive varieties) separately: (1) observed heterozygosity (*H*_*o*_) was obtained as the ratio among heterozygous individuals and the total number of genotypes for each locus; (2) expected heterozygosity (*H*_*e*_) was estimated according to the formula *H*_*e*_ = 1 − ∑*p*_*i*_^2^, where *p*_*i*_ is the frequency of the *i*th allele at the studied locus [25]; (3) the effective number of alleles (*n*_*e*_) was obtained according to the formula (∑*p*_*i*_^2^)^−1^ [26]. In parental population three additional parameters were calculated: (4) probability of identity (PI) was calculated using the formula ∑*p*_*i*_^4^ − ∑∑(2*p*_*i*_ *p*_*j*_)^2^ [27] to show the probability of wrongly assigning a genotype as the pollen donor; (5) exclusion probability (EP) was calculated according to published formula [28], which measures the ability of the marker to exclude a given relationship, and (6) polymorphic information content (PIC) was calculated as 1 − ∑*p*_*i*_^2^ − ∑∑2*p*_*i*_^2^ *p*_*j*_, where *p*_*i*_ equals the frequency of the *i*th allele and *p*_*j*_ the frequency of the (*i* + 1)th allele [29]. *H*_*o*_, *H*_*e*_, PIC, and PI were calculated using the IDENTITY 1.0 program [30] for the analysis of microsatellite data and EP values were computed using FaMoz software [31].

Paternity analysis was performed using the software program FaMoz [31] (http://www.pierroton.inra.fr/genetics/labo/Software/Famoz/index.html). Genotypes of embryos and available genotypes for the 26 reference olive varieties were used to calculate LOD scores (log of the odds ratio or likelihood ratio). These ratios compare the likelihood of an individual being the parent of a given offspring divided by the likelihood of these two individuals being unrelated. FaMoz uses the genotypes of offspring, mother, and potential fathers to calculate the log of the odds ratio scores for any potential parentage relationship [12, 31]. The genotype with the highest LOD score above the threshold value for a given parent/offspring pair is considered the most likely pollen donor [31]. In order to obtain the threshold value of the LOD score to identify true father, a simulation was done using 1000 generated offspring from the genotyped parents. Since mistyping is very likely to occur when scoring microsatellite alleles, the error rate was set to 0.01—both in the simulation and in the assignment of the most likely father [31].

## 3. Results and Discussion

Most of the olive varieties are self-incompatible and need pollinators for successful fertilization and fruit production. In Slovenian orchards the variety “Istrska belica” represents 70% of all olive trees. Growers have noted that there are some existing problems with fruit set and fertility in some growing locations. “Istrska belica” is traditionally believed to be self-compatible, although in the literature it is defined as partly self-compatible [18, 19]. There are no available records or studies regarding the compatibility relationships of this Slovenian olive variety with other potential pollen donors on the molecular level.

Paternity analysis is a method of choice used for paternity determination in plant breeding programs [32]. It is an essential tool for gene and pollen flow studies and for studying the sexual compatibility of plants [33]. In the present investigation this method was used for identifying the most likely pollen donors to the main Slovenian olive variety “Istrska belica” in order to determine the most frequent pollinating varieties and paternal contribution of alleles in monovarietal orchards.

In order to obtain a high quality and sufficient concentration of DNA for further laboratory manipulation, the modified method [14] was used for extraction of DNA from embryos. The main change in the DNA extraction procedure was an extension of precipitation of DNA on ice from 30 minutes to 1.5 hours. Sufficient concentration of DNA was obtained in 31 out of 40 embryos.

Seven microsatellite loci with high polymorphic information content were chosen for genotyping offspring population of embryos. Microsatellites were successfully amplified in all the 31 embryo samples, and a total of 37 different alleles were detected. The average number of alleles per locus was 5.3, and, on average, 2.93 effective alleles per locus were detected (Table 1). The number of amplified alleles at each locus varied from three (UDO99-019) to eight (DCA16). Most of the embryos were heterozygous at locus DCA16 (*H*_*o*_ = 0.903) and the lowest heterozygosity was observed at locus UDO99-019 (*H*_*o*_ = 0.419).

Seven microsatellite loci were more variable in parent than in an offspring population, with the average number of 7.9 alleles per locus ranging from five (UDO99-019) to eleven (DCA9) alleles (Table 2). The highest observed heterozygosity (*H*_*o*_ = 1.000) was found at locus DCA11 and the lowest at locus UDO99-019 (0.654). In the parental population the exclusion probability and probability identity were computed. The cumulative exclusion probability was 0.996, indicating that the set of microsatellite markers was able to exclude almost all unlikely fathers for any given offspring. Similar values of EP were also obtained in paternity in a study of five olive varieties reported by Mookerjee et al. [13] and in a study of sexual compatibility of the olive variety “Kalamata” [12]. The probability of identity (PI) expresses the likelihood of finding two individuals with the same genotype on a defined locus in the population. In most cases PI value was very low, ranging from 0.24 (UDO99-019) to less than 0.05 (DCA3), indicating that the probability to assign an incorrect genotype as the pollen donor was very low. The highest polymorphic information content (PIC = 0.820) was calculated on locus DCA16 and the lowest (PIC = 0.514) on locus UDO99-019. For all loci, except for UDO99-019, PIC values were higher than 0.7, so they are classified as suitable markers for gene mapping.

As expected, calculated genetic parameters were lower in an offspring population than in a parental set of olive varieties due to identical maternal genotype in all embryos. In the parental population, microsatellites loci DCA3, DCA9, and DCA14 detected seven, eleven, and seven alleles, respectively; these results are similar to the number of alleles found by Seifi et al. [12] using the same primer pairs in paternity analysis of the olive variety “Kalamata.”

The most likely method for parentage assignment proposed by Thompson and Meagher [34] is based on the concept of exclusion probability, which is the probability that an unrelated individual will be excluded from being a parent. The likelihood of paternity for each male parent is compared for a particular female parent and her progeny. The logarithm of the likelihood ratios is thus the likelihood of an individual being the parent compared to all other individuals being the parent. Paternity is assigned to the male with the highest likelihood value [14]. In order to determine the potential fathers for the variety “Istrska belica,” genotypes of embryos were compared with genotyping data of 26 reference olive varieties by using FaMoz software [31]. The paternity of the 31 embryos was assigned to the potential pollen donors by calculation of LOD scores. The identification of the threshold value for LOD scores (2.4) was determined at the intersection of curves of two simulations. The first simulation was done on 1,000 randomly generated offspring with father selected at random from a set of genotyped parents, and the second simulation was performed on 1,000 offspring generated according to the randomly associated gametes from allele frequencies in the parental population. True fathers were assigned to the progeny when the LOD score was higher than a threshold (2.4).

For the potential pollen donors the LOD scores ranged from 3.27 to 6.37, with an average of 4.43. The most likely pollen donor was assigned to 12 embryos, while fathers of 8 embryos could not be determined (Table 3). The LOD scores for 8 embryos were lower than the threshold (2.4) determined in the simulations; these are listed as unassigned embryos. For 15 embryos the LOD scores were above the estimated threshold for the paternity (2.4), while 3 of these embryos were assigned with more than one possible pollen donor with the same LOD score. To select the compatible pollinizers, 12 embryos assigned with only one possible pollen donor were used in further analysis.

Varieties “Leccino” and “Leccio del corno” were the most possible fathers for 9.7% of analyzed embryos (Table 4). One possible explanation of this result is that olive trees of the variety “Leccio del corno” which is located very close to the experimental orchard (less than 10 m) has been the main pollinator of mother trees of “Istrska belica.” According to our knowledge there are no trees of the variety “Leccino” located in the immediate vicinity of the experimental field. Nevertheless, “Leccino” and “Leccio del corno” have a common origin in Tuscany, Italy [35], and they probably share some alleles characteristics with Tuscan germplasm. Close genetic relationships between Tuscan olive varieties have been demonstrated in previous microsatellite studies [36, 37]. For 6.5% of embryos, “Picholine” was a major pollen donor, and the varieties “Ascolana tenera,” “Črnica,” “Buga,” and “Grignan” were assigned as possible fathers to 3.2% embryos. These varieties are presented in the Slovene Istria growing region and probably contributed their pollen via the air flows over longer distances. Wind may transport masses of olive pollen grain as far as 12 kilometers from the originating tree [38].

In our study 12 (39%) out of 31 embryos analyzed were assigned to a single father, while potential pollen donors could not be determined for 8 (26%) embryos. Flowers of these samples were probably pollinated by pollen from unidentified genotypes from the nearby olive groves. Similar results were also found in a study of pollination patterns for five olive cultivars in South Australia [13]. The parentage assignment test showed that “Leccio del corno” as well as “Leccino” were the major pollen donors for “Istrska belica.” Similar observation has been reported by Ugrinović and Štampar [18] in the experiment of fertilization of variety “Istrska belica” where “Leccino” was confirmed as the best pollinator for “Istrska belica.” Genotyping analysis of embryos showed that none of the embryos was fertilized with pollen of “Istrska belica.” A very low rate of self-fertility (0.21%) in this variety has also been confirmed by Ugrinović and Štampar [18]. Several studies [11, 33, 39, 40] demonstrated that climatic conditions, especially high temperatures, are the major factor affecting the level of olive self- and crossincompatibility. High temperatures usually have inhibitory effects on self-pollen germination [39], while crosscompatible pollen tubes are less affected [11]. Vuletin Selak et al. [41] have also reported that the self-incompatibility level in olive might be strongly regulated by the temperature; differences in self-fertilization percentage were observed between trees outside and inside polyethylene cages.

## 4. Conclusions

Investigation of the crosscompatibility of olive varieties in Slovene Istria and specifically of “Istrska belica” which is an important variety for Slovenian olive oil production is essential for researchers in order to improve studies examining pollination and fertilization relationships and processes and for growers for optimization of crosspollination, fruit set, and fertility of Slovenian olive orchards. Our study demonstrated that “Istrska belica” is crosscompatible with different olive varieties and the absence of self-fertility requires novel growing guidelines for the growers. The paternity assignment test and FaMoz software used in our investigation proved to be excellent tools for identification of the most likely pollen donors of “Istrska belica.”

## Figures and Tables

**Table 1:** Parameters of genetic variability of each microsatellites locus obtained among offspring population (embryos). Observed (*H*_*o*_) and expected (*H*_*e*_) heterozygosity, number of alleles (*n*), and effective number of alleles (*n*_*e*_).

Locus	*H*_*o*_	*H*_*e*_	*n*	*n*_*e*_
DCA3	0.839	0.682	5	3.14
DCA9	0.710	0.529	5	2.12
DCA11	0.806	0.712	6	3.47
DCA14	0.806	0.698	6	3.31
DCA16	0.903	0.753	8	4.05
UDO99-019	0.419	0.347	3	1.53
EMO-3	0.516	0.656	4	2.91

Average/sum∗	0.714	0.625	5.3/37∗	2.93/20.5∗

**Table 2:** Parameters of genetic variability of each microsatellites locus obtained among parental population (pollen donors). Observed (*H*_*o*_) and expected (*H*_*e*_) heterozygosity, number of alleles (*n*), effective number of alleles (*n*_*e*_), polymorphic information content (PIC), probability of identity (PI), and exclusion probability (EP).

Locus	*H*_*o*_	*H*_*e*_	*n*	*n*_*e*_	PIC	PI	EP
DCA3	0.923	0.844	7	6.41	0.805	0.053	0.590
DCA9	0.885	0.837	11	6.13	0.798	0.055	0.649
DCA11	1.000	0.827	10	5.78	0.786	0.061	0.563
DCA14	0.885	0.754	7	4.07	0.706	0.101	0.533
DCA16	0.923	0.855	9	6.90	0.820	0.045	0.665
UDO99-019	0.654	0.563	5	2.29	0.514	0.239	0.249
EMO-3	0.808	0.725	6	3.64	0.670	0.124	0.438

Average	0.868	0.772	7.9	5.03	0.728	2 × 10^−8^*	0.996∗∗

*Product of PI values for seven microsatellite loci.

**The number represents cumulative value across all 7 loci.

**Table 3:** Number of embryos unassigned, assigned with more than one, and assigned only with one selected pollen donor.

	Number of embryos
Total	31
No likely pollen donor	8
Unassigned	8
Assigned with more than one possible pollen donor (the same LOD score)	3
Assigned with one possible pollen donor	12

**Table 4:** Number of embryos assigned to putative pollen donors in “Istrska belica.”

Pollen donors	Number of embryos
“Arbequina”	0
“Ascolana tenera”	1
“Athena”	0
“Belica Pucer”	0
“Buga”	1
“Cipressino”	0
“Coratina”	0
“Črnica”	1
“Frantoio”	0
“Grignan”	1
“Itrana”	0
“Leccino”	3
“Leccio del corno”	3
“Leccione”	0
“Maurino”	0
“Moraiolo”	0
“Nocelara del belice”	0
“Pendolino”	0
“Picholine”	2
“Samo”	0
“Samo Nova vas”	0
“Santa Catherina”	0
“Štorta”	0
“Zelenjak viseča”	0

Total	12
